# Overview of diet and autoimmune demyelinating optic neuritis: a narrative review

**DOI:** 10.1097/IN9.0000000000000022

**Published:** 2023-04-27

**Authors:** Scott M. Plafker, Tyler Titcomb, Katarzyna Zyla-Jackson, Aneta Kolakowska, Terry Wahls

**Affiliations:** 1 Aging and Metabolism Research Program, Oklahoma Medical Research Foundation, Oklahoma City, OK, USA; 2 Department of Cell Biology, University of Oklahoma Health Sciences Center, Oklahoma City, OK, USA; 3 Department of Internal Medicine, Carver College of Medicine, University of Iowa, Iowa City, IA, USA

**Keywords:** multiple sclerosis, diet, nutrition, optic neuritis, optic nerve, experimental autoimmune encephalomyelitis

## Abstract

This review summarizes the cellular and molecular underpinnings of autoimmune demyelinating optic neuritis (ADON), a common sequela of multiple sclerosis and other demyelinating diseases. We further present nutritional interventions tested for people with multiple sclerosis focusing on strategies that have shown efficacy or associations with disease course and clinical outcomes. We then close by discuss the potential dietary guidance for preventing and/or ameliorating ADON.

## 1. Introduction

Autoimmune diseases have been increasing ^[1,2]^, with 5% to 8% of the US population having one or more of these disorders ^[3]^. This rapid upward trajectory implicates improved diagnostics and changes in environmental risk factors such as diet and lifestyle. The deleterious impacts of diet have in part been attributed to the excessive consumption of ultra-processed foods enriched in simple carbohydrates and pro-inflammatory fats. Consuming ultra-processed foods can cause vitamin and mineral deficiencies ^[4,5]^, disrupt microbiome–host symbiosis, promote systemic inflammation, and drive insulin resistance and metabolic syndrome ^[6–8]^, all of which exacerbate autoimmune sequelae. Moreover, tissue-specific and systemic immune responses are highly integrated with metabolic and gut microbiome status (eg, ^[9–14]^) as the mammalian gastrointestinal tract hosts the largest reservoir of immune cells in the body ^[15]^.

Here, we review the impacts of dietary approaches and components on non-ocular multiple sclerosis (MS) sequelae and consider their applications for MS-autoimmune demyelinating optic neuritis (MS-ADON) based on the candidate mechanisms underlying autoimmune-mediated optic nerve (ON) damage. Ocular pathologies are relatively understudied sequelae of autoimmunity with ADON being the most prevalent visual complication in people with multiple sclerosis (pwMS) ^[16]^, neuromyelitis optica spectrum disorder (NMOSD) ^[17]^, and myelin oligodendrocyte glycoprotein antibody-associated diseases (MOG-AD) ^[18–20]^. ADON affects ~50% of pwMS and is the first demyelinating event (FDE) for ~20% ^[21–23]^. The prevalence of ADON at disease onset for NMOSD and MOG-AD is 50% and 74%, respectively ^[17,24,25]^. Because the most information regarding dietary interventions comes from the MS literature, this review focuses on MS-associated ADON and the potential of nutritional interventions.

ADON results from inflammation and demyelination of the ON, disrupting impulse conduction from the nerve to the visual cortex. Clinical hallmarks include pain, blurred vision, color desaturation, and loss of visual acuity and contrast sensitivity. Axons comprising the ON extend from retinal ganglion cells (RGCs), and demyelination of these axons induces RGC apoptosis. ADON is commonly monocular and lesion localization is retrobulbar in MS ^[26]^. Although most pwMS recover vision following the resolution of inflammation, recurrent flare-ups can culminate in irreversible vision loss. First-line treatment for acute attacks is intravenous steroids, which accelerate recovery for some patients but fail to impact relapse frequency ^[27]^. Second-line treatments include plasma exchange, IV immune globulin, and other agents (reviewed in ^[17,26,28–30]^). Proper diagnosis is paramount as several highly-prescribed MS treatments (eg, glatiramer acetate, interferon β (IFN-β), fingolimod, and dimethyl fumarate) can worsen conditions for people with NMO or MOG-ADs ^[17,31,32]^.

## 2. Cellular and molecular mechanisms of MS-ADON

A brief synopsis follows of the immune cell types, cytokines, and chemokines most associated with MS-ADON, based on the biofluid and immunohistological findings from pwMS and pre-clinical studies. Notably, the pre-clinical studies derive largely from experimental autoimmune encephalomyelitis (EAE), a widely used rodent model for human autoimmune demyelinating diseases of the CNS. Inducible versions of EAE use antigens derived from MOG, myelin basic protein, or proteolipid protein with symptoms modeling numerous motor and visual pathologies of human MS ^[33]^. Transgenic models include (a) the 2D2 mouse that expresses a T-cell receptor for MOG peptide 35–55 and results in age-dependent, isolated optic neuritis ^[34]^; and (b) the OSE opticospinal double strain modeling NMOSD ^[35]^.

### 2.1 IL-17-secreting T cells

IL-17A/F-secreting cells maintain gut homeostasis and communicate gut health throughout the body ^[36]^ but can transition to pathogenicity and promote CNS pathologies in MS and EAE ^[37,38]^. pwMS have increased IL-17 mRNA in their blood and cerebrospinal fluid during relapses ^[39]^. This cytokine is produced by effector T cell types including T_H_17, T_H_1, γδ-T cells, ex-Tregs, CD8^+^ Tc17 cells, natural killer T cells, and group 3 innate lymphoid cells ^[40–43]^. In the context of ADON, adoptively-transferred myelin-specific T_H_17 cells cause more severe optic neuritis and visual deficits than the adoptively-transferred T_H_1 cells ^[44]^, although both cell types drive disease in MS and EAE ^[44,45]^. In fact, IL-17 mRNA expression levels in EAE mice are highest in the ON, compared with the brain and spinal cord, and neutralizing antibodies against IL-17 ablate ADON-mediated structural damage to the ON but are less effective at reducing motor deficits ^[46]^. Additionally, effector T cells on ONs are enriched in markers indicative of T_H_17 transcriptional programming (eg, IL1r and IL23r). γδ-T cells that secrete IL-17 and express the chemokine receptor CCR6 were approximately three-fold higher on the ONs of EAE mice, compared with the brain or spinal cord ^[46]^. Additional pro-inflammatory roles for IL-17 in ADON include recruiting and activating Ly6G^**+**^ neutrophils to the ONs and brains of EAE mice via the subarachnoid space ^[46]^. IL-17 also limits (re)myelination by suppressing the maturation of NG2^+^ oligodendrocyte precursors ^[47]^.

### 2.2 CD8^*+*^T cells

The contributions of CD8^+^ T cells to MS have been reviewed ^[45,48,49]^. Highlights include the following: (a) select major histocompatibility complex (MHC) Class I human leukocyte antigen (HLA) alleles conferring increased or decreased genetic risk for disease (eg, ^[50–53]^); and (b) CD8^+^ T cells being enriched in the CSF ^[54]^ and at lesion edges ^[55]^ and often in greater abundance at sites of infiltrates than CD4^+^ T cells (eg, ^[56,57]^). Contributions of CD8^+^ T cells to ADON have been reported for MS ^[58]^, but more so for NMOSD (eg, ^[58–60]^); however, reduced naïve CD8^+^ T cells and increased effector/memory CD8^+^ T cells in the circulation have been detected for both diseases ^[61]^.

### 2.3 Astrocytes

ON astrocytes play a central role in ADON in the EAE model (eg, ^[62–64]^) with activated astrocytes detectable as an early marker and inducer of inflammation, via NF-кB, on ONs, before frank immune cell infiltration ^[64]^. Elevated lipocalin-2 in EAE studies and patient sera ^[63]^ along with increased complement cascade components, especially C3 and SerpinG1, and suppressed cholesterol biosynthesis machinery were identified as major changes in ON astrocytes contributing to pathogenicity ^[64]^. Curiously, the change in C3 was specific to females and correlated with upregulation of the C3 receptor on microglia/macrophages. These EAE findings fit with C3 knockout mouse studies ^[65,66]^ and complement deposition being detected in MS brain and spinal cord lesions ^[67]^. Reduced cholesterol biosynthesis may underlie compromised remyelination of the ON because astrocytes provide cholesterol to oligodendrocytes for myelin synthesis and to neurons for building synapses and membranes. Fitting with this, enhancing cholesterol transport using gentisic acid ameliorated ON damage and spared vision and ocular structure in EAE mice ^[68]^.

### 2.4 B cells

B cells can present antigen to activate T cells during the earliest stages of ADON, and select B cell-related markers including CD19, CD20, and CD79A in peripheral blood mononuclear cells have been associated with first episodes of ADON. Specifically, higher expression levels of these markers correlated with the severity of visual acuity deficits ^[69,70]^. Interestingly, these markers largely did not differ between subjects irrespective of the extent of vision recovery ^[69]^. In addition, naïve CD19^+^ CD24^+^ CD38^+^ B regulatory cells were higher in people experiencing ADON when compared with age-matched, healthy controls, although differences between the cohorts were not detected in the capacity of these cells to produce IL-10, a primary anti-inflammatory cytokine that suppresses pathogenic T cells through multiple mechanisms ^[71]^.

### 2.5 Macrophages and microglia

Macrophages and microglia are abundant in MS lesions (eg, ^[72–75]^) and blood ^[76]^ and are major drivers of inflammation and pathology early in EAE (eg, ^[72,77–81]^) and in the later stages of disease characterized by gliosis ^[82]^. The involvement of these cell types in ON and retinal inflammation in EAE animals involves NF-кB ^[62]^, the secretion of IL-6 ^[83,84]^, TNF-α ^[85]^, inducible nitric oxide synthase ^[85]^, reactivation within the CNS of auto-reactive T cells, and suppression of tolerance-promoting Tregs (reviewed in ^[86]^). Suppression of macrophage/microglia activation on ONs by inhibition of spermine oxidase ^[87]^ or genetic ablation of arginase 2 ^[88]^ in EAE mice improved visual acuity and reduced macrophage infiltration of ONs. Further, nicotinamide adenine dinucleotide supplementation mitigated microglial and astrocyte numbers on ONs and preserved myelination ^[89]^. In contrast to these pro-inflammatory effector functions of M1-type macrophages, subsets of these cell types (ie, M2-type) are critical for resolving inflammation and promoting repair ^[90,91]^. For example, activated resident microglia promote oligodendrocyte maturation and remyelination ^[92]^, and remove myelin and other cellular debris to assist with regeneration ^[81,93]^.

### 2.6 Infiltrating cell types in ADON lesions and CSF cytokines

The contributions of specific infiltrating cell types to MS-ADON lesions were recently analyzed through immunohistochemical-based immunoprofiling within different lesions (ie, active, chronic active, and inactive) in the parenchyma, meninges, and perivascular regions from post-mortem MS ONs. This study showed that (i) HLA-DR^+^ CD68^+^ myeloid cells were enriched in active lesions and along the edges of chronic active lesions; (ii) CD4^+^ T cells were elevated within chronic active lesions in the parenchyma and within all lesion types in the meninges; (iii) CD8^+^ T cells were detected in various lesions, outnumbered CD4^+^ T cells, but did not reach significance compared with controls; (iv) plasma cells were not enriched in most lesions irrespective of anatomical compartment, but CD138^+^ B cells were lower in active and chronic active parenchymal lesions whereas CD20^+^ B cells were elevated in active perivascular lesions; both markers were increased along the edges of chronic active perivascular lesions. In a study comparing cytokine profiles in the CSF from people experiencing ADON, levels of TNF-α, IL-10, and CXCL13 were higher in those individuals that progressed to a diagnosis of MS as compared to those with isolated optic neuritis that did not progress to MS ^[94]^. The cohort that progressed to MS additionally had increased IgG indices and oligoclonal bands.

## 3. Dietary studies in animal models of MS

Mounting evidence demonstrates profound dietary impacts on EAE, although most studies have not assessed ocular sequelae ^[95–110]^. In those addressing ocular pathology, supplementation with a fatty acid (FA) cocktail of palmitic, oleic, stearic, linoleic, α-linolenic, γ-linolenic, eicosapentaenoic, and docosahexaenoic acids starting the day of MOG_35-55_-EAE immunization counteracted inflammatory and gliotic processes in the retina, reduced multiple inflammatory factors (TNF-α, IL-1β, IL-6, IL-8), and prevented RGC apoptosis ^[111]^. This FA mixture restored the number of myelinated fibers and preserved photopic negative response amplitudes, a readout of inner retinal function. Neuroprotection was attributed to macrophage-derived oncomodulin, a trophic factor for neurons, and to a decreased ratio of pro-inflammatory (M1) to anti-inflammatory (M2) macrophages ^[112]^. Individual FAs also repress disease as shown for conjugated linoleic acid in the OSE model ^[113]^ and valproic acid in EAE rats ^[114]^.

α-lipoic acid (α-LA) dramatically reduced MOG_35-55_-EAE-induced axonal injury in ONs, reduced CD4^+^ T lymphocytes and CD11b^**+**^ macrophages/microglia infiltrates, prevented inner retinal layer thinning, RGC apoptosis, and vision loss whether administered before or after symptom onset ^[115]^. However, a second study showed that α-LA efficacy required administration either before or on the day of MOG immunization to preserve vision and did not prevent ON demyelination or reduce infiltrates ^[116]^. We have shown that a weight-stabilizing ketogenic diet enriched in medium chain triglycerides, flaxseed oil, and insoluble fiber prevents the onset of optic neuritis and motor deficits in MOG_35–55_-EAE mice. When initiated after symptom onset, this diet improved visual and motor function in both male and female mice within 4 days and effectively resolved functional deficits tested within 2 weeks ^[117]^.

## 4. Human MS dietary studies

### 4.1 Dietary impacts on FDEs

**Figure 1.**
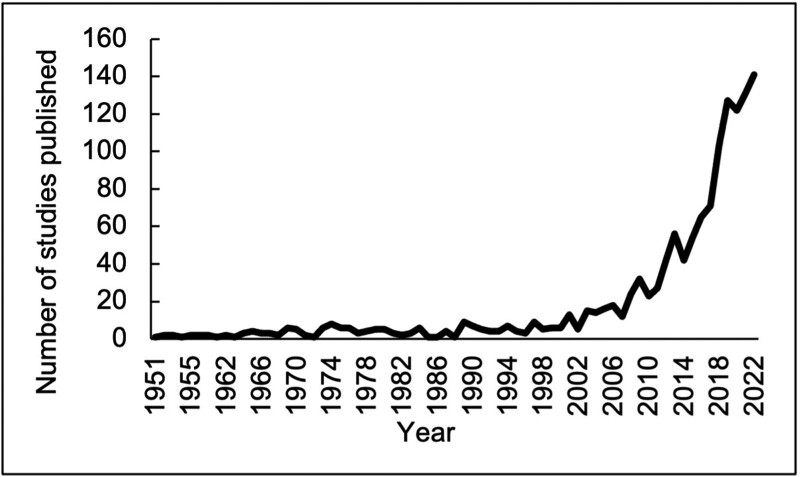
PubMed search results for [Diet AND “multiple sclerosis”] conducted on January 3, 2023. Figure excludes incomplete numbers from 2023.

The intersection of diet and MS has been garnering increased attention (**Figure 1**) but to date, no published human studies have specifically addressed the dietary impacts on ADON risk or resolution. However, studies have investigated the relationship between diet and FDEs, for which ≥20% of cases are expected to be ADON ^[21–23]^. Data from the multi-center, case-control, Ausimmune Study showed that higher yogurt, non-processed red meat, and fish consumption associated with decreased odds of FDE ^[118–120]^. Likewise, the Mediterranean diet and other healthy dietary patterns also associated with reduced odds of FDE in case-control studies ^[121,122]^. In contrast, pro-inflammatory diets (based on the dietary inflammation index ^[123]^) enriched in added sugars and ultra-processed foods are associated with increased FDE odds ^[124]^ and increased risk of autoimmune demyelinating diseases ^[125,126]^. Corroborating these findings, a case-control study reported that low intake of fiber, vitamin D, and α-linolenic acid associated with increased odds of FDE ^[127]^.

### 4.2 Foods associated with improved or worsened MS

The consumption of select foods has been correlated with MS disease trajectory. Higher fiber, fruits, vegetables, and healthy fat intake was positively associated with most health outcomes ^[128]^, and higher consumption of fruits, vegetables, beans, cherries, vitamin D, zinc, vitamin A, calcium, and vitamin B_**6**_ associated with reduced MS risk ^[129–132]^. Higher intake of full-fat dairy associated with lower T2 lesion volumes and higher intake of marine-derived omega-3 FAs aligned with greater normal appearing white matter microstructural integrity ^[133]^. Similarly, a high prudent diet score, consisting of fruits, vegetables, fish, whole grains, and nuts, associated with reduced relapse risk in early MS in a 5-year longitudinal study ^[134]^. A cross-sectional study among 2063 adults with MS revealed a link between higher adherence to Australian dietary guidelines for cardiovascular disease and lower relapse risk, disability, disease activity, and higher quality of life (QoL) ^[135]^. Healthier diet scores also associated with better mental, physical, and total QoL, improved cognitive function, lower depression, anxiety, and pain scores, and fewer cognition, vision, and bowel symptoms ^[136–138]^.

Numerous studies have identified foods and food groups that worsen various clinical outcomes of MS. Higher consumption of beef, butter, pastries, and sweets associated with increased risk ^[130,131]^, whereas not consuming meat correlated with reduced disability progression in pwMS ^[139]^. Diets enriched in added sugars and processed foods corresponded with higher burden of metabolic risk factors including abdominal obesity and reduced circulating high-density lipoprotein concentrations among pwMS ^[140]^. Case-control studies corroborated that pro-inflammatory and low antioxidant-containing diets increased MS risk ^[129,141–144]^.

### 4.3 Dietary patterns that improve MS

Various dietary patterns have been tested in preliminary trials including the low-fat, Mediterranean, Paleolithic, ketogenic, caloric restriction, fast-mimicking, and time-restricted feeding diets. In the 1950s, Dr. Roy Swank reported an association between saturated fat intake and risk of MS ^[145]^. He recommended a low-saturated fat diet to his patients ^[146]^, followed them for up to 50 years ^[147]^, and observed that patients who consumed the least amount of saturated fats were less likely to have disease exacerbations, more likely to retain ambulation, and had reduced risk of mortality ^[148,149]^. Further support for reducing dietary saturated fat comes from a multi-center prospective cohort study in pediatric individuals with MS. This study reported that each 10% increase in energy intake from fat, particularly saturated fat, increased the risk of relapse and each additional one cup equivalent of vegetables decreased relapse risk ^[150]^. Low-fat diets have also been reported to reduce fatigue and improve QoL ^[151–153]^. Curiously, a randomized controlled trial found that a plant-based, low-fat diet did not affect magnetic resonance imaging (MRI) outcomes, relapses, or expanded disability status scale (EDSS) scores over 12 months; however, the control group included an exercise intervention, which confounds this finding ^[153]^.

Numerous MS studies have reported positive impacts of the Mediterranean diet and the Mediterranean-DASH Intervention for Neurodegenerative Delay (MIND). These dietary patterns and other diets with high intake of fruits and vegetables associated with reduced risk of MS ^[130,132,142]^.The Mediterranean diet reduced fatigue and improved QoL in several trials ^[154–159]^, and in a 6-month randomized controlled trial, reduced EDSS progression compared with controls among women with MS ^[155]^. Adherence to the MIND diet associated with thalamic volume in a cross-sectional study among 180 individuals with early MS ^[133]^. Corroborating data from a 5-year longitudinal case-control study among 175 MS cases and 42 controls showed that the AHA Healthy Heart Score diet component inversely associated with 5-year T2 lesion volume accrual ^[160]^.

Multiple dietary patterns with features of ancestral eating patterns have gained attention for pwMS. A multimodal intervention that included a Paleolithic diet led to improved fatigue, QoL, gait, mood, and cholesterol profiles in a single-arm trial ^[161–165]^. Additionally, two randomized controlled trials and one parallel-arm trial confirmed that a modified Paleolithic diet reduces fatigue and improves QoL ^[151,166,167]^. Ketogenic diets yielded similar improvements ^[96,167–169]^. This form of carbohydrate restriction normalizes the colonic microbiome, reduces oxidative stress, inflammation, and serum neurofilament light chain (sNFL) ^[170–172]^. In addition, several calorie-restricted and fasting strategies gave mixed results in preliminary trials with adherence being a primary barrier to conclusive outcomes ^[173–175]^.

### 4.4 Conflicting findings of dietary clinical trials

Not all trials and prospective studies have yielded clear findings to inform dietary guidelines. Most notably, two prospective cohort studies among 56,867 Danish adults and 185,000 US women found no association between diet quality and MS risk ^[176,177]^. The infrequent incidence of MS incidence in both studies (0.2%) may underlie these null findings. In addition, cross-sectional associations of diet quality can be confounded by disability burdens that preclude acquiring and preparing healthy foods ^[178]^. In fact, recent meta-analysis and network meta-analysis of randomized dietary intervention trials found consistent positive effects of several dietary interventions (eg Paleolithic and Mediterranean) on fatigue and QoL; however, due to the small sample size and methodological issues among the preliminary trials, the evidence had limited reliability ^[179,180]^.

## 5. Potential intersections of dietary interventions and MS-ADON

In the absence of clinically-validated nutritional recommendations to advance specific diets that are effective and safe for mitigating MS-ADON, the following section provides dietary guidelines that we posit may prove efficacious based on the multiple mechanisms putatively involved with this ocular sequel: (1) Diets stressing whole foods while reducing or eliminating ultra-processed foods laden with added sugars, additives, hydrogenated fats, and sodium. This recommendation stems from the findings that ultra-processed food consumption associates with increased likelihood of an FDE ^[124,181]^ and with increased MS severity ^[125]^. Furthermore, an abundance of dietary glucose promotes T_H_17 cell differentiation ^[182]^ and auto-reactive T_H_17 cells are implicated as primary drivers of ADON (eg ^[44,46]^). (2) Diets enriched in ω3 anti-inflammatory FAs and limited in ω6 pro-inflammatory FAs. In alignment with the foundational role of inflammation in MS, the types of dietary fats consumed can either positively or negatively impact systemic states of inflammation and gut integrity ^[99]^. For example, ω3 anti-inflammatory FAs, which are enriched in foods like mackerel, salmon, tuna, walnuts, and flaxseed oil, or obtained through supplements, provide precursors for increasing the synthesis of specialized pro-resolving lipid mediators including the resolvins and other endogenous factors that mitigate inflammation. Notably, a meta-analysis of observational studies found that fish consumption is associated with a 23% decreased risk of MS ^[183]^. In contrast, arachidonic acid, select other ω6 FAs, and saturated fats (eg ^[146,147,184]^) should be limited as these lipids fuel the synthesis of pro-inflammatory leukotrienes and prostaglandins ^[185,186]^. Additionally, dietary short-chain FAs, such as propionic acid, have shown benefit in reducing EAE disease severity, whereas dietary long-chain FAs such as lauric acid and palmitic acid (present in coconut oil, palm oil, and soybean oil) exacerbate severity ^[99]^. (3) Diets containing sufficient fermentable and non-fermentable fiber to support a healthy gut microbiome. The commensal microbiota and their collective metabolites are primary regulators of immune cell function, health, and autoimmunity, particularly in the context of T cell-driven autoimmunity in tissues distant from the gut, including the eye ^[187,188]^. Dietary fiber is essential for maintaining a healthy commensal microbiota ^[189–191]^ and low consumption of sufficient fiber has been associated with increased FDE incidence ^[127]^, whereas higher dietary fiber correlates with reduced motor deficits, optic neuritis, and visual acuity loss in EAE mice ^[117,192,193]^ and overall better health outcomes ^[128]^. (4) Dietary patterns that reduce biomarkers of MS disease severity. A recent study ^[171]^ showed that 6 months of an adapted ketogenic diet (ie, average daily intake of >160 g fat [ω6 vs ω3 ratio 2:1], <50 g carbohydrates, and average protein intake ≤100 g/day) reduced sNFL, a recognized biomarker of MS severity ^[194–197]^. Additional clinical and animal evidence support the efficacy of ketogenic diets and other approaches (eg, fast-mimicking diets and intermittent fasting) that induce starvation responses as a common mechanism to ameliorate ADON (eg, ^[117]^ and other diseases [reviewed in ^[198]^]). Recent work has shown that a ketogenic diet can decrease pro-inflammatory T_H_17 cells in the lamina propria, a mucosal layer of the small intestine, and modulate the gut microbiome ^[199]^. However, these strategies may need to be used intermittently (eg, in response to ADON flare-ups) considering long-term compliance is challenging ^[200,201]^. Conflicting results regarding the cardiometabolic impacts of chronic nutritional ketosis also need to be resolved for this approach to gain wider acceptance ^[202,203]^. (5) Identification and elimination of potential autoimmune exacerbating dietary antigens. Sensitivity to dietary antigens is estimated to affect 15% to 20% of the population ^[204]^. Among people with sensitivities, dietary antigens can drive inflammation of the intestinal mucosal layer. However, the link between dietary antigens and MS remains elusive. The modified Paleolithic elimination diet has shown efficacy in clinical trials (eg, ^[151,161,162,165,166,205,206]^) and is based on consuming whole foods while limiting simple sugars, pro-inflammatory fats, as well as gluten, lactose, legumes, nightshades, soy, and whole eggs. pwMS can further optimize this approach by re-introducing select components to identify specific dietary triggers of symptom flare-ups. Additional dietary approaches eliminating antigen-rich foods that presumably drive inflammatory responses have also elicited favorable impacts on MS symptoms (eg, ^[157,207,208]^).

## 6. Conclusions

Disease-specific dietary recommendations are highly desired by pwMS ^[209–212]^. In addition to identifying efficacious dietary strategies, it will be important to develop, in parallel, mechanisms to facilitate adherence. Fortunately, because numerous diets and nutritional approaches have shown benefit in preliminary studies, therapeutic diets may be customizable for each pwMS by taking into account personal and familial dietary preferences, culture, costs, health and metabolic status, and logistical factors such as the ability to acquire and prepare food. The availability of medical foods combined with delivery services will further facilitate access to customized diets, and continuous remote care involving health coaching and wearables (eg continuous glucose monitors) are anticipated to promote adherence.

Word limitations precluded discussing in appropriate detail many diet-related topics relevant to autoimmune demyelination. We refer interested readers to publications covering the central contributions to autoimmunity of FAs ^[14,213]^, gut dysbiosis, and microbiome (eg, ^[214–220]^, and micronutrients ^[11,206,221–225]^).

## Conflicts of interest

The authors declared the following potential conflicts of interest with respect to the research, authorship, and/or publication of this article: Dr Terry Wahls has equity interest in the following companies: Terry Wahls LLC; TZ Press LLC; The Wahls Institute, PLC; FBB Biomed Inc; and the website http://www.terry-wahls.com. She also owns the copyright to the books Minding My Mitochondria (2nd Edition) and The Wahls Protocol, The Wahls Protocol Cooking for Life, and the trademarks The Wahls Protocol and Wahls diet, Wahls Paleo diet, and Wahls Paleo Plus diets. She has completed grant funding from the National Multiple Sclerosis Society for the Dietary Approaches to Treating Multiple Sclerosis Related Fatigue Study. She has financial relationships with BioCeuticals Ltd., MCG Health LLC, Vibrant America LLC, Standard Process Inc., MasterHealth Technologies Inc., Foogal Inc., Genova Diagnostics Inc., and the Institute for Functional Medicine. She receives royalty payments from Penguin Random House. Dr Wahls has conflict of interest management plans in place with the University of Iowa and the Iowa City Veteran’s Affairs Medical Center. All other authors report no personal or financial conflicts of interest in this work.

## Funding

The authors additionally acknowledge the financial support from the Presbyterian Health Foundation (to S.M.P.). T.J.T. is supported by the Carter Chapman Shreve Family Foundation and the Carter Chapman Shreve Fellowship Fund for diet and lifestyle research conducted by the Wahls Research team at the University of Iowa.

## Acknowledgments

The authors acknowledge the support from the National Eye Institute of the National Institutes of Health under Award Number R01EY033782 (to S.M.P.). The content is solely the responsibility of the authors and does not necessarily represent the official views of the National Institutes of Health. In-kind support was provided by the University of Iowa College of Public Health Preventive Intervention Center. Special thanks to KS Plafker for assistance with literature searches and editing
